# Assessment of poultry rearing practices and risk factors of H5N1 and H9N2 virus circulating among backyard chickens and ducks in rural communities

**DOI:** 10.1371/journal.pone.0275852

**Published:** 2022-10-11

**Authors:** Ariful Islam, Shariful Islam, Emama Amin, Shahanaj Shano, Mohammed Abdus Samad, Tahmina Shirin, Mohammad Mahmudul Hassan, Meerjady Sabrina Flora

**Affiliations:** 1 Centre for Integrative Ecology, School of Life and Environmental Science, Deakin University, Australia; 2 EcoHealth Alliance, New York, New York, United States of America; 3 Institute of Epidemiology, Disease Control and Research (IEDCR), Mohakhali, Dhaka, Bangladesh; 4 National Reference Laboratory for Avian Influenza, Bangladesh Livestock Research Institute (BLRI), Savar, Bangladesh; 5 Queensland Alliance for One Health Sciences, School of Veterinary Science, University of Queensland, Queensland, Australia; 6 Directorate General of Health Services, Mohakhali, Dhaka, Bangladesh; West Bengal University of Animal and Fishery Sciences, INDIA

## Abstract

**Background:**

The avian influenza virus (AIV) causes significant economic losses by infecting poultry and occasional spillover to humans. Backyard farms are vulnerable to AIV epidemics due to poor health management and biosecurity practices, threatening rural households’ economic stability and nutrition. We have limited information about the risk factors associated with AIV infection in backyard poultry in Bangladesh. Hence, we conducted a cross-sectional survey comprising epidemiological and anthropological investigations to understand the poultry rearing practices and risk factors of AIV circulation among backyard poultry in selected rural communities.

**Methods:**

We sampled 120 poultry from backyard farms (n = 30) of the three selected communities between February 2017 and January 2018. We tested swab samples for the matrix gene (M gene) followed by H5, H7, and H9 subtypes using real-time reverse transcriptase-polymerase chain reaction (rRT-PCR). We applied multivariable logistic regression for risk factor analysis. Furthermore, we conducted an observational study (42 hours) and informal interviews (n = 30) with backyard farmers to record poultry-raising activities in rural communities.

**Results:**

We detected that 25.2% of the backyard poultry tested positive for AIV, whereas 5% tested positive for H5N1 and 10.8% tested positive for H9N2. Results showed that scavenging in both household garden and other crop fields has higher odds of AIV than scavenging in the household garden (AOR: 24.811; 95% CI: 2.11–292.28), and keeping a cage inside the house has higher odds (AOR:14.5; 95% CI: 1.06–198.51) than keeping it in the veranda, cleaning the cage twice a week or weekly has a higher risk than cleaning daily (AOR: 34.45; 95% CI: 1.04–1139.65), dumping litter or droppings (AOR: 82.80; 95% CI: 3.91–1754.59) and dead birds or wastage (AOR: 109.92, 95% CI: 4.34–2785.29) near water bodies and bushes have a higher risk than burring in the ground, slaughtering and consuming sick birds also had a higher odd of AIV (AOR: 73.45, 95% CI: 1.56–3457.73) than treating the birds. The anthropological investigation revealed that household members had direct contact with the poultry in different ways, including touching, feeding, slaughtering, and contacting poultry feces. Poultry is usually kept inside the house, sick poultry are traditionally slaughtered and eaten, and most poultry raisers do not know that diseases can transmit from backyard poultry to humans.

**Conclusions:**

This study showed the circulation of H5N1 and H9N2 virus in backyard poultry in rural communities; associated with species, scavenging area of the poultry, location of the poultry cage, the practice of litter, wastage, droppings, and dead bird disposal, and practice of handling sick poultry. We suggest improving biosecurity practices in backyard poultry and mass awareness campaigns to reduce incidences of AIV in household-level poultry farms in rural communities in Bangladesh.

## Introduction

Bangladesh has about 1265 people per square kilometer [1], making it one of the most densely inhabited globally [2]. On the other hand, in Bangladesh, 304.17 million poultry are raised, including 255.31 million chickens and 48.86 million ducks yearly [3]. About 80–90% of rural households (HHs) in Bangladesh raise small flocks of poultry in their backyards [4]. Bangladesh relies heavily on poultry and chicken products for its protein needs, consuming an average of 3.2 kilograms of poultry meat and 75 kilograms of eggs annually [5]. On the other hand, inexpensive chicken meat and eggs provide significantly more protein per unit of human intake than cow milk, mutton, pig, and beef. They are expected to be the most consumed animal protein source by 2025 [6]. Poultries are a crucial source of protein. The production of poultry products offers opportunities for poverty reduction, women’s empowerment [7], and improved nutrition for mothers and their young children [8]. Poultry production is also critical to global development in low and middle-income countries (LMICs), such as Bangladesh. It contributes significantly to attaining various sustainable development goals in these nations [9].

The Food and Agriculture Organization of the United Nations classified poultry production systems into four groups based on biosecurity and marketing of birds and their products [10]. Aside from commercial poultry raised in integrated farming systems with moderate to high biosecurity (sector 1–3), a ’backyard’ or ’village level’ poultry industry with lesser biosecurity exists (sector 4) [10]. Native birds or locally accessible breeds are raised in backyards, and the birds or their products are frequently consumed locally. While providing food security to the poorest sections of society, the backyard poultry sector promotes women’s empowerment while having a common environmental effect [11]. Bangladesh is a densely populated country, where about 90% of the rural households rear poultry with humans close interface [12]. It was estimated that approximately 4.4 billion eggs are produced from backyard system poultry production, which covers 67% of the total egg production in Bangladesh [12].

However, backyard farms are in danger of AIV epidemics because of inappropriate risk practices. Along with highly pathogenic avian influenza H5N1, low pathogenic avian influenza H9N2 and many other subtypes have been found in backyard chickens and ducks [13]. On the other hand, Since the start of the H5N1 pandemic in poultry in 2007, Bangladesh has faced a public health and economic threat. With 585 outbreaks recorded in 54 of the 64 districts, the virus has spread enzootically among poultry, making this nation one of those with the most severe outbreaks internationally [14, 15]. In addition, eight instances of H5N1 in humans, one of which resulted in death (4, 9), and three mild cases of H9N2 in humans (10), have been documented in Bangladesh so far. Both H5N1 and H9N2 AIV are now prevalent in poultry and have been seen to infect humans sporadically (11). As part of the avian influenza prevention and control campaign in Bangladesh, the World Health Organization (WHO), the Food and Agriculture Organization (FAO), and the World Organization for Animal Health (OIE) developed a set of 10-step instructions to avoid poultry-to-human transmission [16]. However, very inadequate biosecurity is being maintained in backyard poultry farms.

On the other hand, the highly pathogenic avian influenza A (HPAI) subtype H5N1 is of significant concern globally because of its ability to cause significant morbidity and mortality in birds [17]. H9N2 also has been linked to sickness in birds [18–20]. Other than that, there is also evidence of co-infection of other viral and bacterial viruses in poultry along with H9N2 [21–23]. Previous research data suggest that handling sick and dead poultry, including slaughtering, butchering, and preparing poultry for consumption, contact with the infected blood, body fluids during slaughtering and meat processing, removal of organs, washing meat, feeding, and caring are characterized the primary pathways of transmission of human infection with H5N1 [24–27]. Several indirect factors, including cleaning poultry areas, feces removal, waste using fertilizer, and inhalation also identified as risk factors [28]. Backyard poultry in Bangladesh is vulnerable to AIV infection because chickens are in close contact with domestic ducks and wild birds [29].

Backyard poultry is not fenced, so the birds roam freely from one place to another. Because of the free-range raising systems of backyard poultry, they are more vulnerable to AIV infection. Furthermore, if they become infected, they can transmit the virus to other backyard poultry [30, 31]. Raising poultry species on the same premise and adjacent to sick commercial farmhouse sites increases the risk of infection [32]. It was found in another study that a large number of birds within farm sites, inappropriate biosecurity practices, and the presence of outside birds, especially the Egyptian geese, were associated with increasing the risk of infection [33]. The virus transmission cycle between backyard chickens and ducks would continue until the transmission chain breaks. H5N1 virus in backyard chickens also poses a severe risk to public health because of the frequent and close contact between poultry and humans [29].

Sick poultry slaughtering is a usual practice in Bangladesh [16]. As poultry has economic value to the raisers, changing sick poultry consumption behavior is challenging [16]. The low-income households want to recover their financial loss by consuming sick poultry [16], which is a more critical issue to the raisers other than AIV infection. It was reported from other studies in low-income settings that also have limited practices of standard recommendation or accepting risky strategies, e.g., slaughtering sick poultry, little awareness campaign on avian influenza infection [28, 34].

In Bangladesh, few resources have been available about the risk factors linked with AIV virus infection in backyard poultry. In addition, there are limited data in Bangladesh regarding poultry raising practices and identifying their risk contacts can assist in reducing the risk of avian influenza transmission in backyard farms. However, AIV outbreaks in backyard farms can jeopardize the economic stability of rural households and result in a shortage of nutrition. Hence, this study aimed to investigate the poultry rearing practices and risk factors of AIV circulation among backyard poultry and gain insights into the risk of zoonotic AIV transmission in selected rural communities in Bangladesh.

## Materials and methods

### Study sites, period, and design

**Fig 1:**
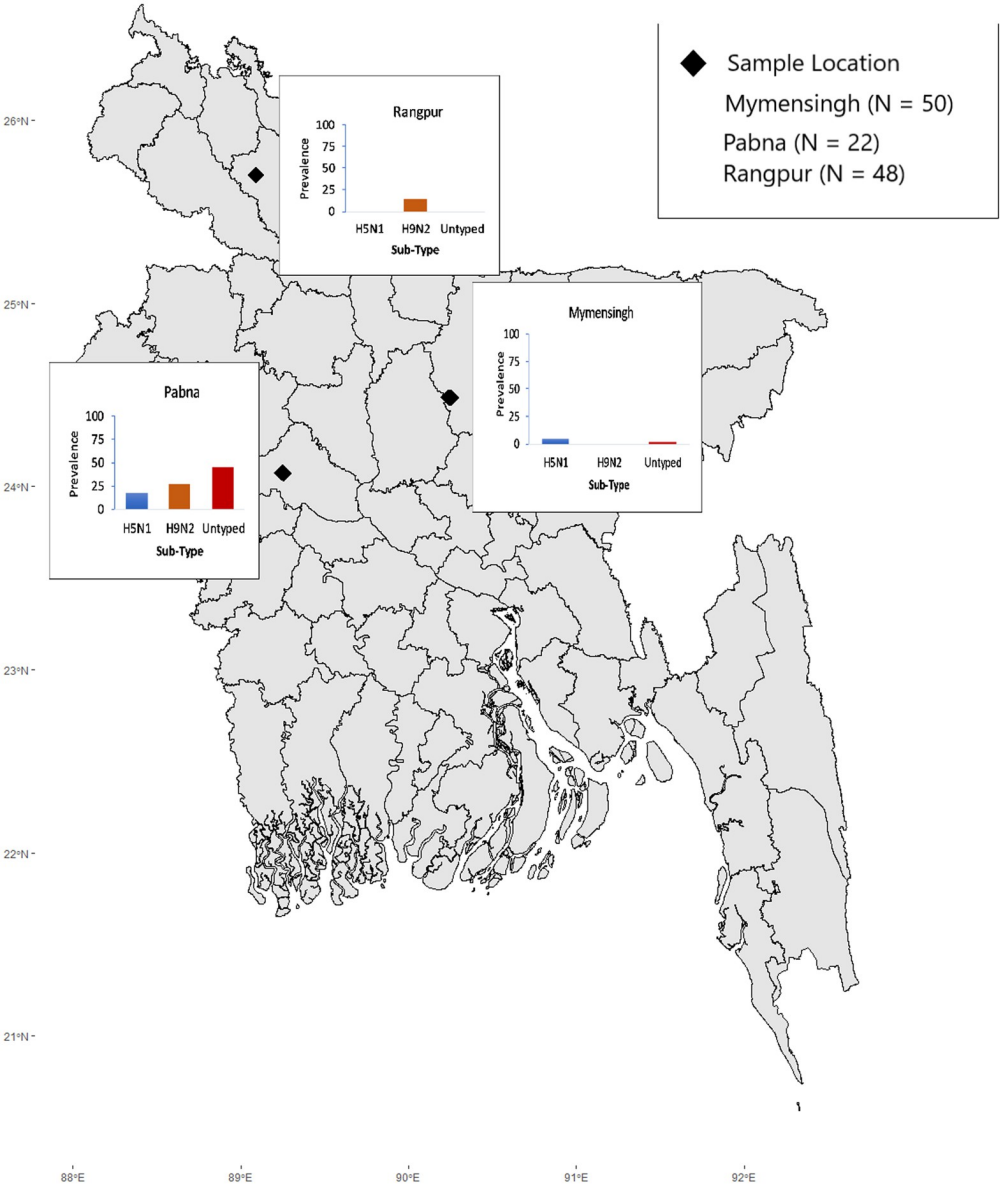
Location of sample and prevalence of H5N1, H9N2, and Influenza A/Untyped.

We conducted a cross-sectional survey comprising epidemiological and anthropological investigations on backyard poultry and their raisers in three villages (Modhupur, Soyaetpur, Malonchi) of the Rangpur, Pabna, and Mymensingh districts from February 2017 to January 2018 (Fig 1). There is extensive commercial layer and broiler farming in Mymensingh. In contrast to Pabna and Rangpur, where backyard farms are more prevalent [3]. In terms of literacy, Pabna has a higher literacy rate than both Rangpur and Mymensingh. On the other hand, Mymensingh and Rangpur have similar income through the agricultural source, which is higher than Pabna. Even though poultry raising practices are identical throughout the country, we selected these three sites to conduct quantitative and qualitative studies in three geographically and socio-culturally distinct places in the country. We purposively selected the villages based on their small size and accessibility.

### Quantitative approach

#### Field sampling procedure

We randomly selected the backyard farms, and from each house, we sampled one bird of each species. With the permission of household owners, we collected the cloacal and oropharyngeal swabs from randomly selected backyard chickens and ducks. The team-maintained PPE (personal protective equipment), including an N95 mask, gloves, and goggles, and physically restrained the birds before sample collection. After collection, both cloacal and oropharyngeal swab samples were placed in a cryotube with 1 mL of Viral Transport Media (VTM). They prepared a single pooled sample (n = 120) preserved in liquid nitrogen in the field and transferred it to the laboratory for storage in a −80°C freezer until further analysis.

#### Data collection

We recorded the necessary information for individual house and bird demographic and farming practices related to biosafety and biosecurity using a structured multiple‐choice questionnaire.

#### Laboratory testing for AIV

We applied the magnetic bead-based RNA isolation technology for *RNA* extraction using the MagMAXTM-96 AI/ND Viral RNA Isolation Kit (Applied Biosystems^™^, San Francisco, CA) in a KingFisher^™^ Flex 96-well robot (Thermo Scientific^™^, Waltham, MA) according to the manufacturer’s procedure. Samples were screened for the presence of AIV by targeting the viral M gene. Firstly, we tested the swab samples for the presence of the M gene using real-time reverse transcription PCR (rRT-PCR) with reference primers and probes, following the procedure described by [35]. Then, we further subtyped the M gene positive samples for the H5, H9, N1, N2, N6, and N8 utilizing primers and probes in an rRT-PCR test [36], followed by E Spackman and DL Suarez [37]. The sample was considered positive if the cycle threshold value was <40 [38]. Among M gene positive samples, those negative for H5, H9, N1, N2, N6, and N8 were considered AIV/untyped.

### Qualitative approach

In the anthropological investigation, we used several qualitative data collection tools, including observation, transect walk, and informal interviews [39]. The trained anthropologist conducted observation to record interactions between humans and backyard poultry with a specific focus on observing feeding, cleaning, touching, hand washing, slaughtering, and waste disposal practices. In doing so, three locations were selected (one in each area) from where they could observe without disturbing the household members. The transect walks technique was performed to identify the households involved with poultry raising. The team led numerous transect walks throughout the community and stopped for informal discussions with community people. Based on the transect walks, the team selected participants involved with poultry raising in their backyard who had knowledge about poultry raising households in their village.

The team conducted 30 informal interviews with members of households, where they conducted observations and other poultry raising households—face-to-face interviews at informants’ preferred time and location, usually in their dwellings. Informal interviews were conducted with the poultry raisers until the team reached data saturation when no new information emerged from different participants [40]. The team built a rapport with the informants to improve the quality of provided information. The team recorded the informal interviews using digital audio recorders. The team also maintained ethnographic diaries to record daily detailed field notes throughout the fieldwork, guiding contextualizing the data.

### Data analysis

#### Quantitative data analysis

The prevalence of Influenza A and its subtypes (H5N1, H9N2, and A/Untype) in sample locations, age, sex, and health status (We assessed the health status of the bird by observing purple discoloration and/or swelling of various body parts; diarrhea; nasal discharge; coughing; and sneezing [41, 42]) was assessed using descriptive statistical techniques. Then, using Pearson’s chi-square tests field [43], associations between chicken and waterfowl rearing practices and Influenza A (Presence or absence of AIV) were calculated, with a p-value of 0.1 being chosen to be included in the model. We then calculated Cramer’s V to identify the association between the explanatory variables [44]. Explanatory variables having Cramer’s V greater than 0.5 are considered to be highly correlated and can be the cause of potential multicollinearity [44]. To investigate associations between the independent factors and the dependent variable and adjust for confounders, a multivariable logistic regression (58) has been used. The logistic regression model provided the adjusted odds ratio (An odds ratio that accounts for other predictor variables in a model is an adjusted odds ratio. In order to account for confounding bias, adjusted ORs are utilized) and 95% CI. Coefficients with p-values (2-sided tests) less than or equal to 0.05 were considered statistically significant in the logistic regression model (5% significance level). We calculated Variance Inflation Factor (VIF) to identify existing multicollinearity in the model [45]. VIF above 3 indicates multicollinearity and is cause for concern [45]. The receiving operating curve’s (ROC) area under the curve (AUC) assesses model performance. All data were analyzed using R (Version 4.1.1, RStudio), and for graphical presentation, GraphPad Prism and STATA (version 16) were also used. We created a map (Fig 1) in Rstudio using a publicly available shape file. The shape file was collected from freely available DIVA-GIS (https://www.diva-gis.org/gdata).

#### Qualitative data analysis

We used the grounded theory approach for the collected themes from the interviewee’s explanations of poultry raising practices. We recorded the interviews that were transcribed verbatim in Bengali. We coded the data manually according to themes and used inductive coding to analyze the materials. We analyzed the qualitative information iteratively and refined the codebook throughout the process to include the additional codes from emergent themes [46].

### Ethical approval

We performed all procedures in studies in accordance with the ethical standards of the institutional and/or national research committee and with the 1964 Helsinki Declaration and its later amendments or comparable ethical standards. The study protocol was approved by the Institutional Ethics Committee of the Institute of Epidemiology Disease Control and Research (IEDCR/IRB/2015/04), Chattogram Veterinary and Animal Sciences University-Animal Experimentation Ethics Committee (protocol: CVASU/Dir (R&E) AEEC/2015/751) and the University of California, Davis (754490–01). As part of standard IRB-approved procedures, participants were informed about the purpose, methods, risks, and benefits of participating in the study and were allowed to ask questions before providing voluntary consent. We obtained informed consent from all study participants before conducting interviews.

## Results

### The overall prevalence of AIV and subtypes in backyard poultry

The prevalence of AIV was 25% in the studied backyard poultry samples. A total of 5% (95% CI: 2.3–10.8) of the poultry had H5N1 influenza, whereas 10.8% had H9N2 (95% CI: 6.4–17.9).

### Spatial prevalence of AIV in the study area

The prevalence of AIV for Mymensingh, Pabna, and Rangpur was 6%, 90.91%, and 14.54%, respectively (Fig 1). We detected H5N1 (18.2%), H9N2 (27.3%), and Influenza A/Untyped (45.5%) in samples from Pabna; however, H5N1 (4%) and Influenza A/Untyped (2%) were detected in poultry from Mymensingh and only H9N2 (14.6%) in Rangpur community.

### Prevalence of AIV and subtypes in sex, age and health status

**Fig 2:**
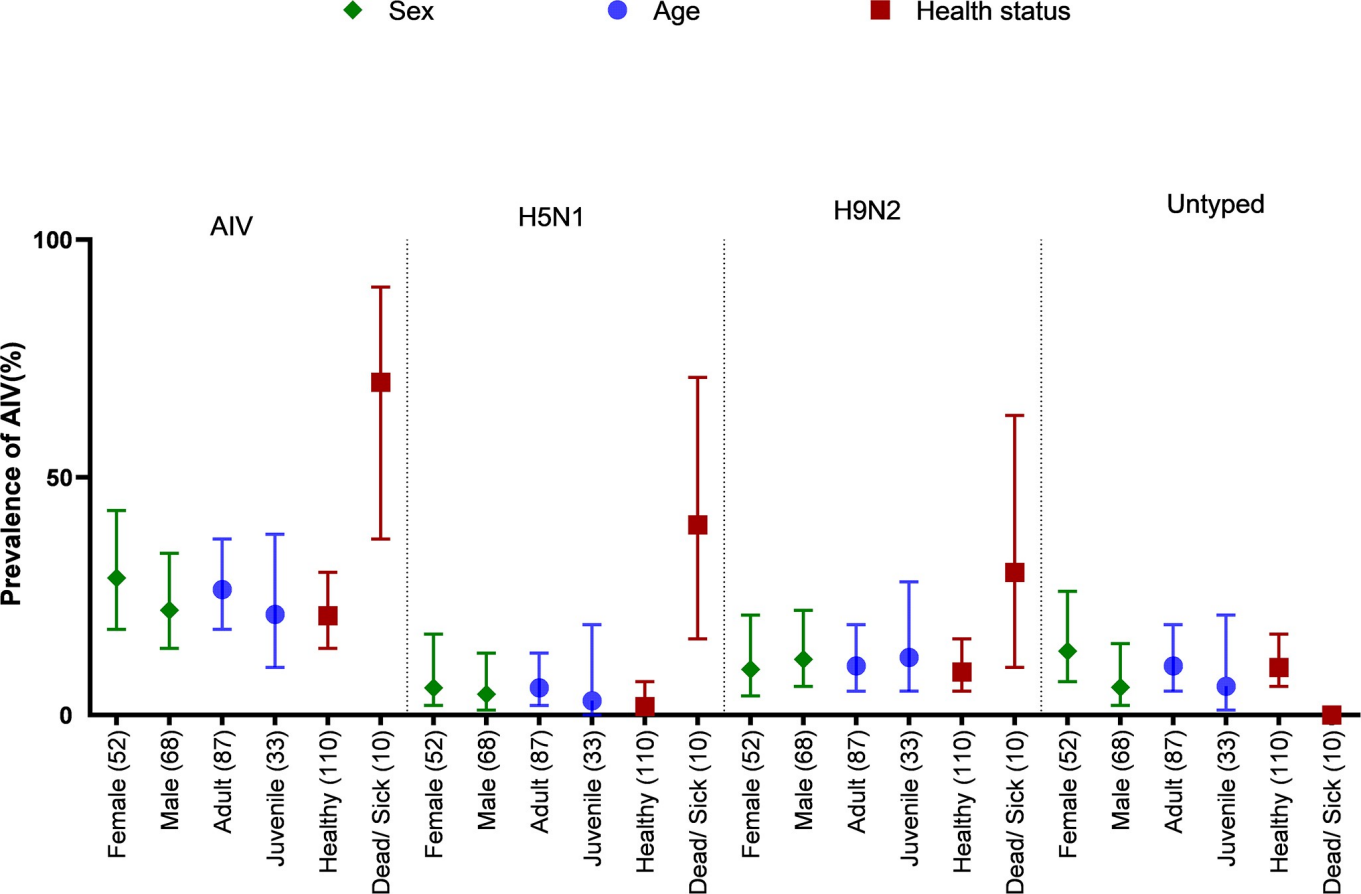
Prevalence and 95% CI of Influenza A, H5N1, H9N2, and Influenza A/Untyped in sub-samples (sex, age, health status).

The prevalence of Influenza A and sub-types, along with 95% CI by sex, age, and health status, has been presented in Fig 2. The prevalence of influenza A was higher in females (28.85%, 18–43) than in males (22.1%, 95% CI: 14–34). Similarly, females (5.8%, 95% CI: 2–17) had a greater prevalence of H5N1 than males (4.4%, 95% CI: 1–13), and the same was true for Influenza A/Untyped; female (13.5%, 95% CI: 7–26) and male (5.9%, 95% CI: 2–15). Nevertheless, the prevalence of H9N2 was higher in males (11.8%, 95% CI: 6–22) than in females (9.6%, 95% CI: 4–21).

On the other hand, the prevalence of Influenza was higher in adult (26%, 95% CI: 18–37) chickens and waterfowl than in juveniles (21.2%, 95% CI: 10–38). Similarly, Adult poultry (5.8%, 95% CI: 2–13) had a higher H5N1 prevalence than young poultry (3%, 95% CI: 0–19). Influenza A/untyped showed a similar distribution pattern, predominately in adult poultry (10.3%, 95% CI: 5–19) and young poultry (6.1%, 95% CI: 1–21). Although the prevalence of H9N2 was higher in juvenile poultries (12.1%, 95% CI: 5–28) than in adults, the prevalence of H9N2 in juvenile poultries was lower in adults (10.3%, 9% CI: 5–19).

In health status, AIV prevalence was recorded higher in dead/sick poultry (70%, 95% CI: 37–90) than in apparently healthy poultries (20.9%, 95% CI: 14–30). Similarly, the prevalence of H5N1 and H9N2 were also observed higher in dead/sick poultries (H5N1: 40%, 95% CI: 16–71; H9N2: 30%, 95% CI: 10–63) than in healthy poultries (H5N1: 1.8%, 95% CI: 0–7; H9N2: 9.1%, 95% CI: 5–16).

### Univariate analysis to identify the chicken and waterfowl rearing practices that are associated with Influenza A

Species, Flock size, scavenging areas, presence and or access of wild birds around the backyard, location of poultry cage, cleaning frequency of cage, disposal of litter and or droppings, the practice of dead birds and wastage disposal, the practice of handling with sick bird, presence of Rodent were the significant rearing practices associated with Influenza A in chicken and waterfowl (Table 1).

**Table 1:** Chicken and waterfowl rearing practices associated with Influenza A (Results to chi-square test).

Variable	Category	AIVc n (%)	95% CI	p-value
Species	Chicken	22 (42.31)	(30,56)	<0.001*
	Waterfowl	8 (11.67)	(6,22)	
Flock size	0–10	17 (19.54)	(12,29)	0.025*
	>10	13 (39.39)	(24,57)	
Feed source	Both (commercial and homemade)	8 (38.1)	(20,60)	0.302
	Commercial feed	4 (20)	(8,43)	
	Homemade	18 (20)	(15,33)	
Scavenging areas	Both areas (household garden and other crop fields)	20 (20)	(50,83)	<0.001*
	Household garden	10 (10.99)	(6,19)	
Other livestock and peri domestic animals’ presence	No	12 (21.43)	(13,34)	0.398
	Yes	18 (28.13)	(18,40)	
Source of young poultry	Home hen incubate	17 (28.33)	(18,41)	0.399
	Nearby market	13 (21.67)	(13,34)	
Presence and or access of wild birds around the backyard	No	6 (11.32)	(5,23)	0.002*
	Yes	24 (35.82)	(25,48)	
Location of poultry-cage	Inside house	17 (32.69)	(21,47)	0.089*
	Yard/veranda	13 (19.12)	(11,30)	
Cleaning frequency of cage	Daily	8 (13.33)	(7,25)	0.003*
	Twice a week or weekly	22 (36.67)	(25,50)	
Slaughter	No	7 (17.07)	(8,32)	0.149
	Yes	23 (29.11)	(20,40)	
Disposal of litter and or droppings	Dumped in a pit and used as fertilizer	5 (8.2)	(3,18)	<0.001*
	Throw away nearby water bodies or bushes	25 (42.37)	(30,55)	
Practice of dead birds and wastage disposal	Buried on ground	5 (7.94)	(3,18)	<0.001*
	Throw away nearby water bodies or bushes	25 (43.86)	(32,57)	
Scavenging near water bodies	No	11 (19.64)	(11,32)	0.205
	Yes	19 (29.69)	(20,42)	
The practice of handling sick bird	Isolating and treatment	2 (4.76)	(1,17)	0.001*
	Sell	10 (34.48)	(20,53)	
	Slaughtering and consume	31 (63.27)	(24,51)	
Same cage for both species	No	11 (26.19)	(15,42)	0.825
	Yes	19 (24.36)	(16,35)	
Presence of Rodent	No	9 (13.24)	(7,24)	0.001*
	Yes	21 (40.37)	(28,54)	
Raise more than one species	No	6 (16.67)	(8,33)	0.168
	Yes	24 (28.57)	(20,39)	

* P-value<0.1, Statistically Significant, CI- Confidence Interval

In our sample, households with chickens had a higher prevalence of influenza A (42.31%, 95% CI 30–56) than those with waterfowls (11.67%, 95% CI: 6–22). Households with larger flock sizes (flock size >10) had a higher prevalence of being AIV positive than those with smaller flock sizes (flock size < = 10). Households with chicken and waterfowl scavenging in the household garden and other crop fields had almost double the prevalence of those with chicken and waterfowl scavenging just in the household garden (10.99%, 95% CI: 6–19).

Influenza A was more common in households with the presence or access of wild birds around the backyard (35.82%, 95% CI: 25–48) than in those who did not (11.32%, 95% (CI: 5–23).

Households who keep their poultry cages inside the house had a higher prevalence (32.69%, 95% CI: 21–47) of Influenza A positive chicken and waterfowl than those who keep their poultry cages in the yard/veranda (19.12%, 95% CI: 11–30). The household that cleaned the cages daily had a much lower prevalence of AIV (13.33%, 95% CI: 7–25) than those who cleaned twice a week or weekly (36.67%, 95% CI: 25–50).

A higher percentage of Influenza A was detected among those households who throw away litter or droppings in nearby bushes and water bodies (42.37%, 95% CI: 30–55) than those who dump in a pit and use it as fertilizer (7.94%, 95% CI: 3–18).

Prevalence of Influenza A was highest among the households that slaughter and consume sick chicken and waterfowl (63.21%, 95% CI: 24–51). In contrast, 34.48% (95% CI: 20–53) of Influenza A was detected in the households that sell sick chicken and waterfowl. The prevalence of influenza was lowest in the households that isolate and treat their chicken and waterfowl (4.76%, 95% CI: 1–16).

Households with rodent presence have a significantly higher prevalence of Influenza A (40.37%, 95% CI: 28–54) than those without the presence of rodents (13.24%, 95% CI: 7–24).

### Exploring correlation between predictor variables to identify potential multicollinearity

**Fig 3:**
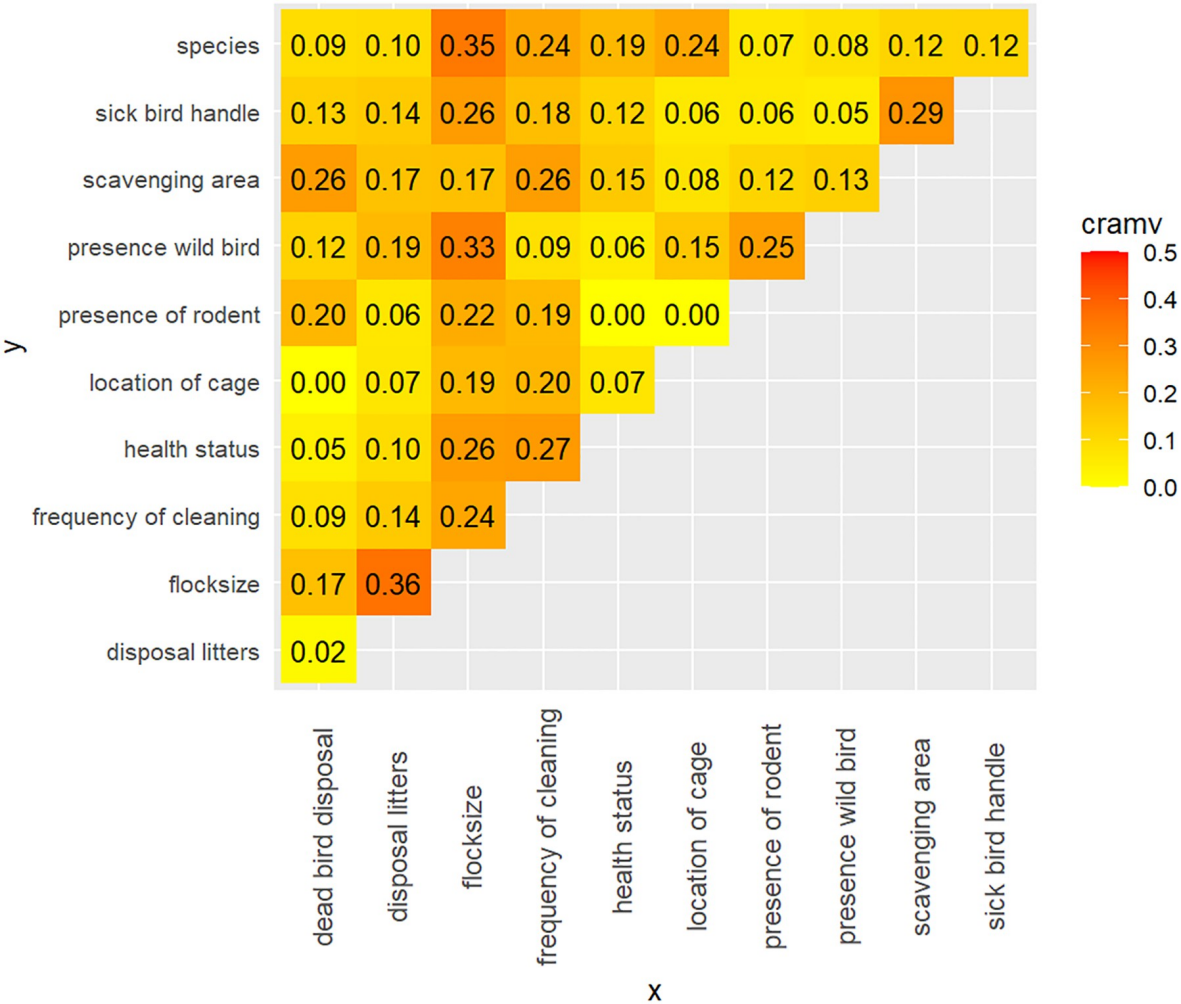
Association between the predictor variables using a matrix of Cramer’s V value.

The value of Cramer’s V between the predictor variables is shown in Fig 3. A greater Cramer’s V value suggests a stronger association. We chose 0.50 as the cutoff value [44]. We can see that none of our explanatory variables has Creamer V > 0.50. So, we included all the explanatory variables identified in the univariate analysis in the multivariable model.

### Multi-variable logistic regression to identify the relationship between chicken and waterfowl rearing practices and Influenza A

The estimated effects from the logistic regression model for the factors associated with Influenza A are presented in Table 2.

**Table 2:** Relationship between chicken and waterfowl rearing practices and Influenza A (Results to multivariable logistic regression model).

Variable	Category	AOR	95% CI	P-value
Species	Waterfowl	1.00	-	-
	Chicken	44.33	(1.67, 1183.88)	0.024*
Flock size	< = 10	1.00	-	-
	>10	14.98	(.90, 249.73)	0.059
Scavenging area	Household garden	1.00	-	-
	Both areas (household garden and other crop fields)	24.811	(2.11, 292.28)	0.011*
Presence of wild bird	No	1.00	-	-
	Yes	17.48	(.92, 333.99)	0.057
Location of poultry-cage	Yard/veranda	1.00	-	-
	Inside house	14.5	(1.06, 198.51)	0.045*
Cleaning frequency of poultry-cage	Daily	1.00	-	-
	Twice a week or weekly	34.45	(1.04, 1139.65)	0.047*
Disposal of litters or droppings	Dumped in a pit and used as fertilizer	1.00	-	-
	Throw away nearby water bodies or bushes	82.80	(3.91, 1754.69)	0.005*
Disposal of dead birds and wastage	Buried on ground	1.00	-	-
	Throw away nearby bodies or bushes	109.92	(4.34, 2785.29)	0.004*
The practice of handling the sick bird	Isolating and treatment	1.00	-	-
	Sell	64.43	(.74, 5647.47)	0.068
	Slaughtering and consume	73.45	(1.56, 3457.73)	0.029*
Presence of rodent	No	1.00	-	-
	Yes	3.14	(.37, 26.91)	0.296

*P-value<0.05, Statistically Significant; CI- Confidence Interval; AOR- Adjusted Odds Ratio

It demonstrates that out of 10 factors from Pearson’s chi-square test, only seven factors remain significant (at a 5% significant level) after fitting the logistic regression. Species, scavenging area, location of poultry cage, cleaning frequency of poultry cage, Disposal of litters or droppings, the practice of dead birds and wastage disposal, and the practice of handling sick birds remain significant factors in Influenza A.

In our sample, households with chickens have 44.33 times higher odds of being Influenza A positive than those with waterfowl. Those Households whose chicken and poultry scavenge in both household garden and other crop fields have a much higher odds (AOR: 24.81; 95% CI: 2.12–292.28) of being Influenza A positive than those whose chicken and waterfowls scavenge only in the household garden.

Household with poultry cages in the yard/veranda has a 93% less chance of being Influenza A positive than those with poultry cages inside the house; if the poultry cages are cleaned twice a week or weekly, then the possibility of being Influenza A positive increased 34.45 times then those households who clean poultry cages daily.

Households that throw away the litter and droppings in nearby water bodies or bushes have a much higher odds (AOR: 82.80, 95% CI: 3.907–1754.687) of being Influenza A positive than those who dumped the litter or droppings in a pit. Disposal of dead birds and waste had a similar result as disposal of litter and droppings. Households that throw away the dead birds and waste in nearby water bodies or bushes are much more likely to be Influenza positive than those households that dump the dead birds and waste in a pit. Though the odds of being Influenza A positive in households that isolate and treat their sick birds were not significantly different from those that sell the dead birds, households that slaughter and consume the sick birds had a higher chance of being Influenza A positive (AOR: 73.45, 95% CI: 1.560–3457.731).

### Variance inflation factor (VIF) and Receiver operating curve (ROC) for measuring model performance

The value of the mean VIF for our multivariable logistic model is 2.06. As VIF < 3.00, it indicates that there is no potential multicollinearity exists in our model.

**Fig 4:**
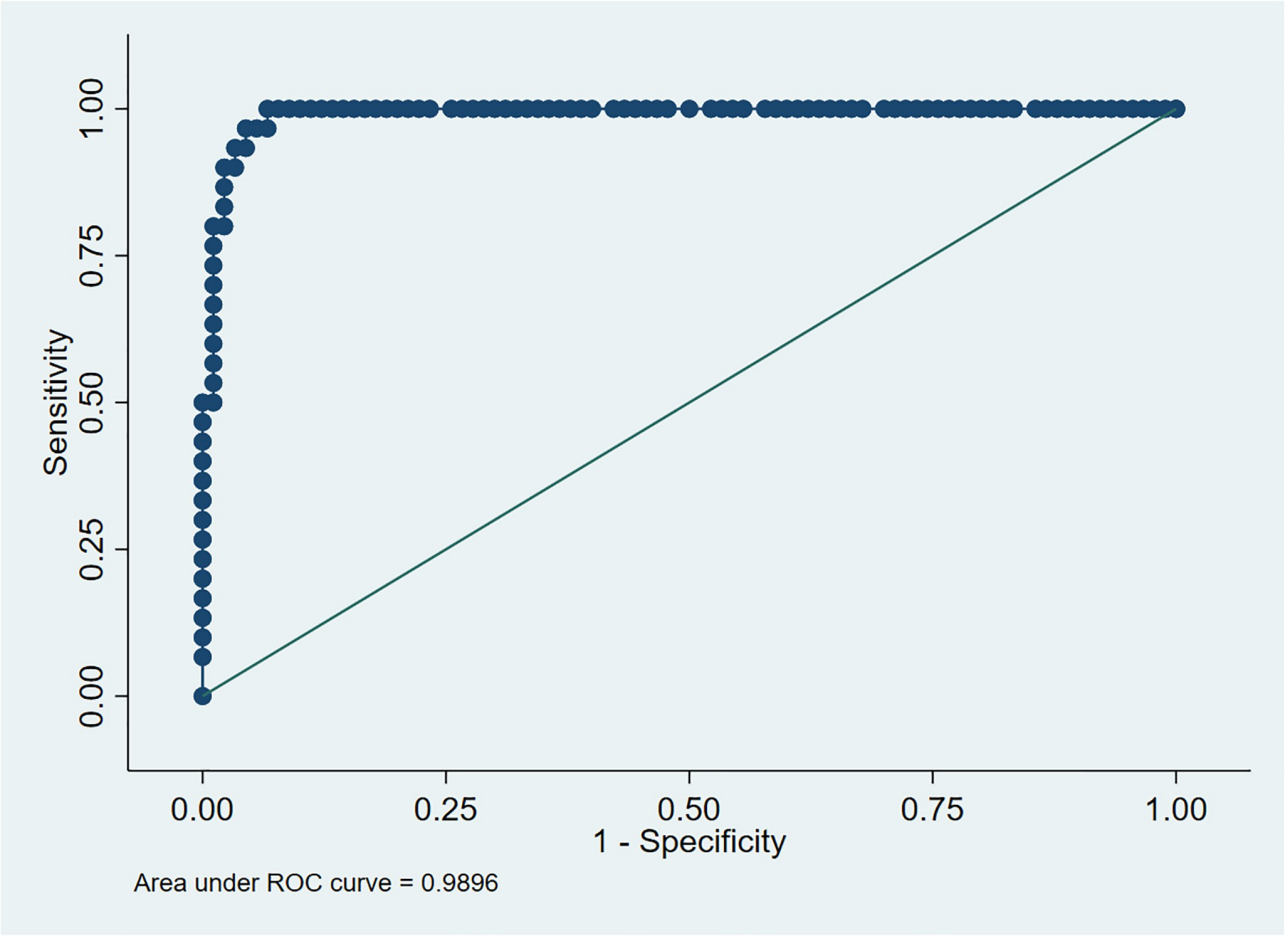
Performance measure for predicting Influenza A using last fitted multivariable logistic regression model.

The receiver operating characteristic (ROC) curve’s area under the curve (AUC) has been calculated to measure model performance, which evaluates sensitivity vs. specificity and explains the model’s performance. Fig 4 shows that the final fitted model has a 98.7% chance of correctly distinguishing between the positive and negative Influenza A classes. This indicates that the inferences drawn from our model are appropriate.

### Qualitative findings

#### Interactions between humans, poultry, and other animals

Household members have direct and indirect contact with chicken and duck as part of regular raising practices. People generally encounter poultry by touching, feeding, slaughtering, and contacting the feces in different ways, including handling while cleaning the shed and household premises, walking on feces with bare feet, and communicating with roaming poultry inside the household premises. These practices had been performed from generation to generation, and interestingly, they never thought these traditional raising practices could lead to disease transmission to humans from poultry.

Most (73.3%, 22/30) households in three areas kept their poultry inside their bedroom at night, observed during physical observation. A few of the respondents (26%, 8/30) from informal interviews also reported that they kept poultry within the kitchen, bamboo, or wooden fenced area, located in the kitchen or next to their yard or corridor. Poultry raisers preferred to keep poultry in their kitchens and bedrooms because they feared potential poultry theft from the outside and feared snatching poultry by wild animals such as foxes or dogs.

*“If I keep my poultry outside the home*, *I could lose them*. *Thief could take them”*.

*(Informant from Rangpur)*

*“I do not have separate space to keep them outside*. *It is costly to make a shed outside”*.

*(Informant from Mymensingh)*

Another common practice was that domestic chicken and duck were kept in the same shed in the backyard in all three areas. Lack of space was the main reason to keep poultry and goose together, and other causes include not knowing if chicken and duck could transmit diseases/viruses from each other as they are different species. It was also informed that building a separate shed for poultry is quite expensive.

*“We do not have a separate shed to keep duck and chicken*, *and we keep those within hour kitchen room under the bamboo cage*.*”*

*(Informant from Mymensingh)*

It was observed that poultry scavenges both inside and outside the house, frequently in the kitchen, bedroom, yard, and corridor. Moreover, backyard poultry scavenges near commercial farms, where disease can spread. Often poultry goes to the dining area during mealtime with the family members. During the observation, it was observed that poultry bites on the plate where rice was kept for lunch, and one family member usually removed rice from the biting area and gave it to another family member to eat.

*“We do not keep poultry in the cage for daytime*. *They can roam everywhere at home”*.

*(Informant from Pabna)*

All respondents were directly involved in the rearing and feeding of poultry hatching eggs. Informants reported that they rarely used soap after handling, feeding, cleaning sheds, and slaughtering poultry, and they walked barefooted during poultry rearing activities. It was also found that wild birds like Common myna, Asian pied starling, and wild animals, including dogs entered the house and shared food with the backyard poultry. Usually, backyard poultry raisers provide food to their poultry in the open space, and at that time, other birds and wild animals visit and share food with the poultry.

Most people (93.3%, 28/30) throw poultry offal and guts in nearby household places and water bodies like ponds and rivers. It was observed that they also threw the dead chicken in nearby areas. It was found from the informal interviews that, at the village level, they do not have any station to dispose of the waste regularly produced in their household. So, they dispose of garbage in places where they feel convenient. Therefore, they also dispose of poultry waste in the same place.

*There are no separate places for waste dumping*. *So*, *we dispose of waste in personal open spaces*, *ponds*, *or canals*.

*(Informant from Mymensingh)*

Slaughter their sick poultry for household consumption; sometimes, they sold also sick poultry. They slaughter their sick poultry without personal protective equipment, including gloves and masks. Moreover, village people do not have any designated places for slaughtering activities.

#### Poultry raiser’s perception among zoonotic diseases like bird flu

Most informants (76.66%, 23/30) were unaware of any diseases that might transmit from poultry to humans. Most of the informants from all three sites did not hear about avian influenza or bird flu. Few of them said they had heard about bird flu, but they had not seen any infected people with the bird flu. As a result, they doubt if this disease could transmit to humans from poultry. Most of the participants in Mymensingh heard about bird flu as there were many commercial poultry farms. his might affect information, education, and information campaigns in different media.

*I have seen the bird flu on TV that can spread from poultry to poultry and humans*. *However*, *I have not seen anyone who has been infected with this flu*. *Maybe nobody is infected in our country*.

*(Respondent from Rangpur)*

A widespread perception reported by the poultry rearing communities is that sick poultry could be consumed because all germs or harmful objects are killed and destroyed by the heat of fire during cooking. Another critical perception is that the disease only spreads from poultry to poultry, not poultry to humans.

#### Health care-seeking practices for poultry

Most of the raisers (83.33%, 25/30) from all three sites mentioned that they use home remedies for their poultry, including feeding onion, which was a common practice for caring for sick poultry in rural communities. The raisers also reported other practices, such as feeding warm rice, warm water, and juice of herbal leaves. If the condition of the poultry does not improve, they go to a nearby pharmacy where they can get medicines easily. Several raisers from the Rangpur said that they usually seek to care for their poultry from the local veterinary care providers. Local care providers provide free advice and sell medicines to raisers. Typically, these local care providers have medicine shops in the village market, and sometimes they keep medicine in their homes.

*I do not go to the doctor to take medicines for my chicken*. *I go to the doctor when my goat or cow becomes sick*. *Either I take treatment from local doctors or go to government facilities*. *I feed them onion with rice for my chicken when I find them sick*. *If I feel they will not be cured*, *then I slaughter them for self-consumption*, *and I sell the rest of them in the market*.

*(Respondent from Mymensingh)*

While taking medicines from the local pharmacy, they usually describe the poultry’s signs and symptoms to the shopkeeper or local care provider. People from three study sites said that they rarely go to government veterinary hospital facilities to take the treatment for poultry due to the long-distance and availability of doctors. People could easily reach them, or local care providers could visit raisers’ households. Moreover, several informants said they slaughter poultry when they see any sickness symptoms. Most informants think that it is better to consume sick poultry rather than make them waste.

#### Market and value chain of backyard poultry in the community

We found in all three communities that women were mainly involved in raising household ducks and chickens. Women can earn money by selling ducks and chickens, spending on household expenditures, and saving money for future investments. Community people can consume eggs and poultry from their flock, which is the primary protein source for families in rural areas. A few raisers (23.33%, 7/30) mentioned that they usually slaughter their poultry for household consumption when the duck and chicken are sick. Moreover, they slaughter poultry when they entertain their guests or during any festival.

*In my home*, *I raise chicken and duck*. *We can have eggs and meat when I need them*. *I slaughter a chicken when I cannot buy meat from the market*.

*(Respondent from Pabna)*

It was found from all three study sites that they usually try to sell their chicken and duck in the nearby market. Sometimes they return the unsold poultry from the market, which is exposed to other poultry. It was reported from the Fulbaria that sometimes poultry traders come into the household to buy chicken and duck. Sometimes neighbors come to the home to purchase chicken and duck. Most poultry raisers (90%, 27/30) have been informed that they hatch chicken and duck from their flock; sometimes, they buy from other poultry raisers. Few of them buy from the market.

Moreover, community people buy broiler chicken from the nearby local market for protein consumption. Several people mentioned that sometimes they buy live broiler poultry and slaughter them after bringing the house. Few community people said they want to avoid buying from the market because they think that chicken and duck could be sick when they buy from the market. According to them, if poultry remains sick, sickness could transmit to their flock.

## Discussion

The rural environment is friendly for backyard poultry raising, and poultry raising provides critical support to the family’s livelihood. Still, this increases the risk of avian influenza because of its ability to infect poultry, and this virus has consistently had a negative economic effect on Bangladesh’s poultry industries. It has also remained a potential concern in human health [16, 47, 48]. Commercial poultry farms and live bird markets have been the primary targets of epidemiological monitoring and research into avian influenza (AI) in Bangladesh [29, 49, 50]. There are a few AIV research on backyard poultry raising systems available. This research examined the poultry rearing practices that may influence avian influenza and the risk of zoonotic transmission.

Ducks and chicken raising provide a source of protein [51] and an additional source of income to the rural community people. This additional income generated by women from backyard poultry can increase nutrition and provide economic support in various ways [16]. However, it also comes with the risk of disease transmission if proper bio-security measures are not taken. According to the findings of this study, backyard poultry flocks are not exempted from avian influenza exposure. Studies by [52, 53] also found AIV in backyard poultry in Bangladesh. In addition, we found that chickens and waterfowl raised in backyards have been exposed to multiple subtypes of AIV. Similar findings were found in other studies in Bangladesh [52]. We also found A/H5 circulating in the backyard poultries in two of our study sites, which can be concerning as HPAIV H5N1 can transmit from poultry to humans and cause mortality.

When comparing the dead birds with the healthy birds, we found a higher prevalence of AIV and its subtypes (A/H5 and A/H9). Other studies have reported findings that are similar to these. Even though they obtained much fewer samples from sick and dead birds, HPAIV was at higher levels in dead birds [54]. Furthermore, we found a higher prevalence of AIV, A/H5N1, and A/Untyped viruses in adult poultry. This may be because adult poultries can roam more freely than young poultries and are exposed to a greater variety of wild birds and animals. This was also reported in the qualitative part of the current study.

A greater risk of influenza was detected in poultry that scavenged both household gardens and other crop fields than in poultry that only scavenged in the household garden. Most backyard poultries in Bangladesh scavenge both inside and outside the house (scavenging in nearby crop fields or neighboring households). The backyard poultries in Bangladesh scavenge in the yard, veranda (A veranda is a roofed, open-air porch attached to the outside of the house), cattle shed, surrounding bushes, neighboring households, and nearby crop fields [16]. Scavenging in surrounding crop fields raises the danger of infection from other animals and humans.

We found that having poultry cages inside the house rather than outside or on the veranda increased the likelihood of AIV. Limited space and poor air circulation within the place allow the virus to persist for extended periods. However, most raisers (55%) keep their birds inside their homes in Bangladesh, while others keep them outside in a coop made of soil or wood with a tin roof, and these practices are aligned with the previous study [16]. Moreover, keeping poultry in the bedroom [55] and exposure to feces have suggested potential risks to AIV in humans [16]. Furthermore, we found that influenza decreases if the poultry cages are cleaned daily. Nevertheless, in Bangladesh, farmers who kept birds in cages cleaned them every two to four days [56], and this practice was observed in the qualitative interviews in the current study.

Throwing away dead birds, wastage, litter, and droppings in open places, water bodies, or near human settlements is responsible for spreading influenza. Similar results were found in other studies [57]. Moreover, in most areas in Bangladesh, offal and viscera are dumped into nearby water bodies and shrubs [16]. On the other hand, almost all sick birds are slaughtered for human consumption [56]. The findings of the qualitative interviews of the current study showed that people try to isolate themselves first and provide treatment from the local veterinary medicine shops, which might decrease the risk of AIV. However, if the birds are not cured, they try to sell (to neighbors or markets) or slaughter them for self-consumption.

We also found that poultry raisers closely interacted with their poultry, including touching poultry, feeding sick poultry by hand, walking on feces barefoot [58, 59], and de-feathering and slaughtering poultry. Women seem at high risk of disease transmission since they are involved in direct contact through numerous interactions, including poultry raising, de-feathering, and butchering, which increases the risk of poultry-to-human transmission.

Our results also show that poultry sheds are made of locally available materials, including clay, mud, bamboo, and wood. Similar findings have been identified in other studies [16, 60, 61]. They also showed that backyard poultry feeding costs are nominal, as duck and chicken foraged freely for feed. Taking medical care for household duck and chicken, raisers have chosen local poultry care providers over government service providers because local providers lived closer, were accessible throughout the day, and distributed advice and medication upon request [61]. Moreover, our results suggest that household duck and chicken raisers trust local care providers as reliable treatment sources rather than government facilities. Furthermore, poultry raisers slaughter their poultry for consumption if the poultry’s health deteriorates. Sick poultry slaughtering activities are common in Indonesia and China [62–64].

To sell villagers’ poultry, they go to the market, and sometimes middlemen come to the village and buy poultry. These practices are also found in other studies in Bangladesh [65] and Pakistan [66]. Moreover, poultry raisers add new poultry, hatch from their flock, and purchase from other poultry raisers and live bird markets [67].

Backyard poultry raisers in Bangladesh have minimum knowledge of avian influenza or bird flu, and government messages may not have reached the receivers in rural Bangladesh. Up to the community, people will believe that avian influenza could affect them; they are unwilling to change the hands-on approaches they have been practicing for many years [16]. Such practices have been observed in the current study, which could increase the risk of infection.

We could not gather samples from all country areas due to a lack of resources and funds. Collecting data from a regular surveillance program will be necessary to make a more precise inference. Virus DNA sequencing can aid in knowing the molecular evaluation of viruses present in Bangladesh’s backyard farms and the identification of new viruses.

## Conclusion

This study highlighted that both HPAIV H5N1 and LPAIV H9N2 were circulating in backyard farms of Bangladesh. We identified risk factors related to poultry bird characteristics, farm management, and farm biosecurity characteristics that contribute to the outbreak of avian influenza in the Backyard poultry farms of Bangladesh. This study pinpoints that applying simple biosecurity measures such as constraining the birds scavenging area in their household or keeping the bird in the yard instead of inside the house can prevent the spread of avian influenza. This study provides a baseline for similar studies in Bangladesh and other developing countries in the future. Good management and strict biosecurity can prevent AIV. Management of identified risk factors is a crucial consideration to mitigate the future risks of AIV. For this, a surveillance and contingency plan for AIV should be prepared and implemented to effectively control and contain AIV in Bangladesh. This will ultimately contribute to minimizing socioeconomic losses due to AIV outbreaks. We suggest a detailed analytic study on this in the future taking more farms and other geographic areas of Bangladesh.

## Supporting information

S1 Data (CSV)

S1 File (DOCX)

S2 File (DOCX)
