# Supplementary material for: Assessment of poultry rearing practices and risk factors of H5N1 and H9N2 virus circulating among backyard chickens and ducks in rural communities
Source: PLoS One. 2022 Oct 11;17(10):e0275852. doi: 10.1371/journal.pone.0275852 (PMC9553037; doi:10.1371/journal.pone.0275852)
Supplement: S1 File — (DOCX) [file pone.0275852.s002.docx]

**Checklist for Informal Interview**

**Section A: Demographic Information**

Place of discussion:

Gender:

Age:

Education:

Occupation:

Monthly income (approximate):

**Section B: Human, Poultry and other animal interactions:**

1. Generally, how do you come into contact with the poultry in regular basis? (Probe- by touching, feeding, slaughtering, and making contact with the feces in different ways, hatching eggs). What do you do after touching them to make your hand clean? (washing hands with soap)
2. In general, what are some places where your poultry birds graze?
3. What is the accommodation arrangements do make to keep the poultry birds during night? What are the reasons for such (based on the response) arrangements?
4. Do you keep multiple species in same shed? Why?
5. Do your poultry come into contact with the foods you or your family members consume? What do you do with those food if come into contact?
6. How do you feed your poultry? (come into contact with other wildlife during feeding)
7. Where do you dispose offal and poultry waste? Do you have any dedicated waste disposal place? (Location of the place).
8. What do you do with your sick poultry? {[probe: Slaughter /sell, using protective gear during handling sick poultry], ask ‘why’ to each behavior}.

**Section C- Health care seeking practice for poultry**

1. What do you do if your poultry birds get sick? (Probe: Consult to a Veterinarian, local animal health care providers, and local drug shop, apply traditional healing methods). What are the factors that influence for taking a particular treatment method?

**Section D: Market and Value chain of backyard poultry**

1. What are some purposes that motivate you to raise poultry birds? (Meat consumption, selling, egg production: consumption/selling, hatch chicken and duck from their flock, earn money)
2. Where do you sell your poultry and egg? (Nearby village market, neighbors)? From where do you buy poultry for raising purpose? (market, neighbors: reasons behind those)

**Section E: Zoonotic disease perception among poultry raisers**

1. Do you know about any diseases that could spread from poultry birds to human? [What are those, how these diseases could be spread?]
2. Have you heard of any zoonotic disease? [what are these diseases, source of information]
