# Supplementary material for: Assessment of poultry rearing practices and risk factors of H5N1 and H9N2 virus circulating among backyard chickens and ducks in rural communities
Source: PLoS One. 2022 Oct 11;17(10):e0275852. doi: 10.1371/journal.pone.0275852 (PMC9553037; doi:10.1371/journal.pone.0275852)
Supplement: S2 File — (DOCX) [file pone.0275852.s003.docx]

**Government of The People’s Republic of Bangladesh**

**Institute of Epidemiology, Disease control and Research (IEDCR)**

**Mohakhali, Dhaka-1212**

**Questionnaire:** *Household farm Biosecurity Practices*

**Date:**

| **A.** | **Farmer’s demography** | | | | |
| --- | --- | --- | --- | --- | --- |
| 1. | ID: | | | | |
| 2. | Contact number: | | GPS | Lat: | Long: |
| 3. | Village: | Union: | | | |
| 4. | Upazila: | District: | | | |
| **B.** | **Farm Characteristics** | | | | |
| 5. | Types of poultry: | Chicken/ Duck/ Both Chicken and Duck/ Mixed:/others: | | | |
| 6. | Size of the flock | Chicken……………Duck………………Others………… | | | |
| 7. | Average age of the poultry:  (in days) today | Cock……………Hen……………Drake……………Duck…….…  Chicks…………………………Ducklings………………………... | | | |
| 8. | Source of your chick/ducklings | Chicken: Nearby Farm/Local LBM / Incubate by Brooding Hen/ Neighboring hatchery/ Neighbor Others:  Ducks: Nearby Farm/Local LBM / Incubate by Brooding Hen/ Neighboring hatchery/ Others | | | |
| **C.** | **Management and biosecurity** | | | | |
| **Housing and feeding management** | | | | | |
| 9. | Location of bird’s house: | | Chicken: within the yard/ outside the yard/ dedicated place/ within the living room  Duck: within the yard/ outside the yard/ dedicated place/ within the living room | | |
| 10. | How frequently change or clean the litter/droppings/waste | | 1. Once a day 2. Once a week 3. Twice a week 4. Once a month 5. Other specify ------ | | |
| 11. | How do you dispose the droppings and/or litter? (multiple answer) | | 1. Throw on nearby bushes 2. Compost in dustbin/ large pit and used as fertilizer in field 3. Throw away in nearby waterbodies 4. Left in the yard 5. Others: | | |
| 12. | What types of feed supplied to your birds? (multiple answer) | | 1. Chicken: Homemade (rice bran/whole rice/rice polish/cooked rice/paddy) Commercial feed/Grains/No specific feed other than scavenging/other (specify)------- 2. Duck: Homemade (rice bran/whole rice/rice polish/cooked rice/paddy) /Crushed snail/ Commercial feed/Grains /No specific feed other than scavenging/ other (specify)------- | | |
|  |  | |  | | |
| 13. | Do you feed different species of poultry in a same feeder/trough/space? | | 1. Yes 2. No 3. No specific feeder other than spreading the feed on the floor space 4. NA (if single species rear) | | |
| 14. | How Frequently do you are cleaning poultry shed/cage? | | 1. . …..times/day 2. . …..times/week 3. . …..times/month | | |
| **Biosecurity practices** | | | | | |
| 15. | when your farm affected by disease, what do you do with birds not sick yet | | a) Sell local market  b) slaughter and eat  c)Treatment  d) Other specify--------- | | |
| 16. | Do you keep duck and chicken together in the same house at night? | | 1. Yes 2. No 3. NA (if single species reared) | | |
| 17. | What do you do with dead birds? | | 1. Buried/burned 2. Throwing on nearby open places or bushes 3. Throwing on nearby waterbodies (ponds or river) 4. Fish feed 5. Others: | | |
| 18. | Vaccinated against AIV: | | Yes, time of last dose:  No | | |
| 19. | Do your poultry mix with neighbor backyard waterfowls during scavenging? | | Yes  No | | |
| 20. | Can stray dogs and or cats mix with poultry during feeding? | | Yes  No | | |
| 21. | Do your chicken have access to mix and feeding with wild birds? | | Yes  No | | |
| 23. | Have you seen any vermin and/or rodent access to your poultry yard/ house? | | Yes  No | | |
